# Exploring the complexity and spectrum of racial/ethnic disparities in colon cancer management

**DOI:** 10.1186/s12939-023-01883-w

**Published:** 2023-04-14

**Authors:** Anya L. Greenberg, Nathan R. Brand, Alan Zambeli-Ljepović, Katherine E. Barnes, Sy Han Chiou, Kim F. Rhoads, Mohamed A. Adam, Ankit Sarin

**Affiliations:** 1 Department of Surgery, University of California San Francisco, 550 16Th Street, 6Th Floor, San Francisco, CA 94158 USA; 2 Helen Diller Family Comprehensive Cancer Center, San Francisco, CA USA

**Keywords:** Colon cancer, Colorectal surgery, Racial disparities, Healthcare access

## Abstract

**Background:**

Colorectal cancer is a leading cause of morbidity and mortality across U.S. racial/ethnic groups. Existing studies often focus on a particular race/ethnicity or single domain within the care continuum. Granular exploration of disparities among different racial/ethnic groups *across the entire colon cancer care continuum* is needed. We aimed to characterize differences in colon cancer outcomes by race/ethnicity across each stage of the care continuum.

**Methods:**

We used the 2010–2017 National Cancer Database to examine differences in outcomes by race/ethnicity across six domains: clinical stage at presentation; timing of surgery; access to minimally invasive surgery; post-operative outcomes; utilization of chemotherapy; and cumulative incidence of death. Analysis was via multivariable logistic or median regression, with select demographics, hospital factors, and treatment details as covariates.

**Results:**

326,003 patients (49.6% female, 24.0% non-White, including 12.7% Black, 6.1% Hispanic/Spanish, 1.3% East Asian, 0.9% Southeast Asian, 0.4% South Asian, 0.3% AIAE, and 0.2% NHOPI) met inclusion criteria. Relative to non-Hispanic White patients: Southeast Asian (OR 1.39, *p* < 0.01), Hispanic/Spanish (OR 1.11 *p* < 0.01), and Black (OR 1.09, *p* < 0.01) patients had increased odds of presenting with advanced clinical stage. Southeast Asian (OR 1.37, *p* < 0.01), East Asian (OR 1.27, *p* = 0.05), Hispanic/Spanish (OR 1.05 *p* = 0.02), and Black (OR 1.05, *p* < 0.01) patients had increased odds of advanced pathologic stage. Black patients had increased odds of experiencing a surgical delay (OR 1.33, *p* < 0.01); receiving non-robotic surgery (OR 1.12, *p* < 0.01); having post-surgical complications (OR 1.29, *p* < 0.01); initiating chemotherapy more than 90 days post-surgery (OR 1.24, *p* < 0.01); and omitting chemotherapy altogether (OR 1.12, *p* = 0.05). Black patients had significantly higher cumulative incidence of death at every pathologic stage relative to non-Hispanic White patients when adjusting for non-modifiable patient factors (*p* < 0.05, all stages), but these differences were no longer statistically significant when also adjusting for modifiable factors such as insurance status and income.

**Conclusions:**

Non-White patients disproportionately experience advanced stage at presentation. Disparities for Black patients are seen across the entire colon cancer care continuum. Targeted interventions may be appropriate for some groups; however, major system-level transformation is needed to address disparities experienced by Black patients.

**Supplementary Information:**

The online version contains supplementary material available at 10.1186/s12939-023-01883-w.

## Introduction

Colorectal cancer is a leading cause of morbidity and mortality in the U.S [1]. Disparities in colorectal cancer diagnosis, treatment, and survival are well established for U.S. racial/ethnic minorities relative to non-Hispanic White patients. Black patients have an increased risk of developing and dying from colorectal cancer [2], and this gap has widened as the reduction in mortality has lagged [3, 4]. Hispanic patients have been shown to have lower screening rates [5, 6] and present with later-stage disease [7], and do not equally benefit from new treatments [8]. Asian patients have the lowest odds amongst ethnic groups in the U.S. of having a primary care physician (PCP) and have been noted to have concerningly low rates of screening [9, 10]. While reasons for these disparities are multifactorial, patient-specific factors such as health beliefs, language discordance, and health literacy, as well as system-level barriers in accessing care, such as insurance, geography, and healthcare services capacity, have been cited as key contributors variably impacting each of these groups [2, 10–17].

Although numerous studies have highlighted these racial/ethnic disparities, many focus only on disparities in a particular domain within the care continuum (e.g., screening, treatment delays, survival) or for a particular race/ethnicity [5, 7, 9, 11, 12, 14]. Additionally, existing studies often lack granularity, characterizing racial/ethnic groups broadly (e.g., “Asian,” rather than differentiating between Southeast Asian, East Asian, etc.). However, as these domains frequently have downstream or additive effects and the US has continued to become increasingly diverse [18], a nuanced understanding of this complex issue is critical for effective intervention.

We aimed to address this gap by performing an in-depth exploration of colon cancer care using the National Cancer Database (NCDB). Specifically, we characterized differences in colon cancer outcomes by race/ethnicity, using as detailed definitions of race/ethnicity as the NCDB would allow, across six stages of the care continuum. We used this characterization to paint a comprehensive picture of disparities in colon cancer presentation and management, propose potential reasons for the disparities seen in the context of our findings and existing literature, and identify areas that may warrant intervention. Although the root cause of racial/ethnic disparities in the healthcare system cannot be addressed without macro-level change, identifying specific groups that face disparities in each domain of the care continuum may provide opportunities for targeted interventions.

## Methods

We retrospectively analyzed data for the years 2010–2017 from the National Cancer Database (NCDB). The NCDB includes data on facility factors, patient demographics, tumor characteristics and staging information, treatment details, and outcomes from more than 1,500 hospitals, accounting for 70% of new cancer cases in the U.S [19]. This study was approved by the Institutional Review Board at the University of California—San Francisco (UCSF): Study Number 18–26,677.

### Patient population

All adult patients ≥ age 18 who had surgery for their first malignant or in situ primary tumor of the colon (including right, transverse, left, and rectosigmoid colon) between 2010 and 2017 were included. Patients who received neoadjuvant chemotherapy or radiation therapy and those who underwent concurrent surgeries (e.g., distant resection during primary surgery) were excluded. This inclusion/exclusion criteria was established to hone in on patients who received surgery with curative intent given the well-established standard of care for this patient population.

### Independent variables

Select patient demographics, facility factors, and treatment details were included as covariates in our analysis. Patient demographics included NCDB-defined age category, sex, race/ethnicity, insurance, income quartile, high school degree quartile, whether the patient was from a metropolitan, urban or rural county, distance patient traveled to receive care, whether the patient is from a Medicaid expansion state, and Charlson-Deyo comorbidity score [20].

The NCDB estimates household income and educational attainment by matching each patient’s zip code to the American Community Survey data, which provides median household income and the number of adults who did not graduate from high school by zip code. Both measures are categorized based on equally proportioned quartiles among all U.S. zip codes. Using this approach, income quartiles were defined as < $40,227 (Q1), $40,227-$50,353 (Q2), $50,354-$63,332 (Q3), and >  = $63,333 (Q4) median income and high school degree quartiles were defined as >  = 17.6% (Q1), 10.9%-17.5% (Q2), 6.3%-10.8% (Q3), and < 6.3% (Q4) did not graduate from high school [21].

A combined race/ethnicity variable was developed for our analysis. Individuals reported to be of Hispanic/Spanish origin in the NCDB were given this classification in our dataset (i.e., Hispanic/Spanish ethnicity, all races). For all other patients, the Race category from the NCDB was used (i.e., non-Hispanic/White, non-Hispanic/Black, non-Hispanic/American Indian, Aleutian, and Eskimo (AIAE), Native Hawaiian and other Pacific Islander (NHOPI), etc.). Asian race categories (i.e., those patients with NCDB Race codes 04–08, 10–17, 20–22, 25–28, 30–32, and 96–97) were grouped as East Asian, Southeast Asian, South Asian, and Native Hawaiian and Other Pacific Islander based on the definitions from the U.S. Census Bureau [22, 23] and Asian Pacific Institute on Gender-Based Violence [24].

Facility factors included in our analyses were facility type (academic, community cancer program, comprehensive community cancer program, and integrated network cancer program) and facility location (i.e., region of the U.S.). Treatment details included in our analysis were the year of diagnosis, operative approach (open, laparoscopic, or robotic), and tumor location.

### Outcomes

We examined outcomes along the continuum of colon cancer management. This included six domains: 1) clinical stage at presentation; 2) timing of surgery; 3) access to minimally invasive surgery (MIS); 4) post-operative outcomes; 5) utilization of chemotherapy; and 6) cumulative incidence of death.

The first domain, clinical stage at presentation, was examined for patients who had clinical (and pathological) stage available. The second domain, timing of surgery, included three subdomains that are related to the timing of the initial treatment: time from diagnosis to first surgery (where > 42 days was considered to be a delay in surgery based on prior literature in this area) [25], pathologic stage, and upstaging between presentation and surgery. To examine upstaging between presentation and surgery, only patients with both clinical and pathologic stage available were included. The third domain, access to MIS, assessed surgical approach (i.e., open, laparoscopic or robotic procedure). The fourth domain is post-operative outcomes, a composite variable created as a proxy for the presence of postoperative complications based on available outcome measures in the NCDB. This composite variable reflected the occurrence of any of the following outcome measures: post-procedure hospital length of stay (LOS) > 7 days, readmission within 30-days, or mortality within 30-days. The fifth domain, utilization of chemotherapy, was examined for all surgical patients with pathologic stage III or IV who would have met National Comprehensive Cancer Network® (NCCN) criteria for recommendation of adjuvant chemotherapy [26]. Specifically, rates of chemotherapy recommendation, omission (and reasons for omission), and delays greater than 90 days were assessed. Finally, the sixth domain, cumulative incidence of death, was examined using the date and vital status at time of last patient contact. In multivariable modeling, this domain was examined in two ways: with covariates including non-modifiable factors (sex, age, geography, comorbidities, year of diagnosis, and tumor location) and with covariates including both non-modifiable factors and modifiable factors (insurance, income quartile, education quartile, urban or rural county, facility type, distance traveled, and surgical approach). This was done to gauge whether structural interventions may be impactful.

Outcome variables from each domain were included, where appropriate, as covariates in analyses of subsequent domains to adjust for potential confounding related to differences in outcomes within prior domains.

### Statistical methods

Descriptive statistics were used to characterize patient factors. Counts and percentages are reported for nominal data. Unadjusted comparisons of outcomes of interest for all independent variables were performed using the Chi-square test for categorical variables or the Kruskal–Wallis test for continuous variables, followed by multivariable logistic or median regression, with select demographics, facility factors, and treatment details as covariates. Missing data were excluded from the analysis, however, multiple imputation was applied to assess for material differences in results. Hypothesis tests were two-sided, and the significance threshold was set to 0.05. Statistical analyses were performed using SAS version 9.4.

## Results

For the study period of 2010–2017, 326,003 patients (49.6% female and 24.0% non-White, including 12.7% Black, 6.1% Hispanic/Spanish, 1.3% East Asian, 0.9% Southeast Asian, 0.4% South Asian, 0.3% AIAE, and 0.2% NHOPI) met our inclusion criteria (Table 1).

**Table 1:** Patient Demographics, Facility Factors, and Treatment Details of Study Population

	**Surgical Patients**	**Surgical Patients with Pathologic & Clinical Stage Available**	**Surgical Patients with Pathologic Stage III/IV**
**Characteristic**	**n (%** ^a^ **)**	**n (%** ^a^ **)**	**n (%** ^a^ **)**
**All Patients**	**326,003**	**125,508**	**123,670**
Patient Demographics			
**Age Category**			
18–49	37,404 (11.5)	14,118 (11.2)	16,361 (13.2)
50–59	66,662 (20.4)	26,467 (21.1)	25,825 (20.9)
60–69	82,827 (25.4)	32,267 (25.7)	31,202 (25.2)
70–79	76,569 (23.5)	29,465 (23.5)	27,266 (22)
80 +	62,541 (19.2)	23,191 (18.5)	23,016 (18.6)
**Sex**			
Female	161,636 (49.6)	62,900 (50.1)	61,471 (49.7)
Male	164,367 (50.4)	62,608 (49.9)	62,199 (50.3)
**Race/Ethnicity**			
White	247,688 (76)	95,813 (76.3)	92,162 (74.5)
American Indian, Aleutian, and Eskimo	1,048 (0.3)	399 (0.3)	397 (0.3)
East Asian	4,191 (1.3)	1,513 (1.2)	1,775 (1.4)
Native Hawaiian and Other Pacific Islander	531 (0.2)	166 (0.1)	232 (0.2)
Other Asian	3,027 (0.9)	1,071 (0.9)	1,183 (1)
South Asian	1,310 (0.4)	475 (0.4)	485 (0.4)
Southeast Asian	2,820 (0.9)	1,026 (0.8)	1,282 (1)
Black	41,278 (12.7)	16,255 (13)	16,720 (13.5)
Hispanic/Spanish	19,974 (6.1)	7,313 (5.8)	8,010 (6.5)
Other	1,856 (0.6)	665 (0.5)	690 (0.6)
**Payor**			
Commercial	120,390 (36.9)	46,628 (37.2)	46,231 (37.4)
Medicaid	19,149 (5.9)	7,439 (5.9)	8,269 (6.7)
Medicare	167,973 (51.5)	63,792 (50.8)	61,163 (49.5)
Other Government	3,065 (0.9)	1,117 (0.9)	1,174 (0.9)
Uninsured	10,826 (3.3)	4,450 (3.5)	5,014 (4.1)
**Income Quartile**			
Q1	53,378 (16.4)	21,060 (16.8)	21,474 (17.4)
Q2	78,783 (24.2)	30,781 (24.5)	29,904 (24.2)
Q3	68,611 (21)	27,411 (21.8)	26,509 (21.4)
Q4	94,952 (29.1)	36,633 (29.2)	35,155 (28.4)
**High School Degree Quartile**			
Q1	67,715 (20.8)	25,934 (20.7)	25,072 (20.3)
Q2	77,853 (23.9)	31,453 (25.1)	30,186 (24.4)
Q3	95,547 (29.3)	37,446 (29.8)	35,897 (29)
Q4	54,786 (16.8)	21,128 (16.8)	21,952 (17.8)
**County**			
Metro	274,085 (84.1)	105,520 (84.1)	103,287 (83.5)
Urban	39,782 (12.2)	15,235 (12.1)	15,541 (12.6)
Rural	5,581 (1.7)	2,026 (1.6)	2,245 (1.8)
**Distance Traveled**			
< 12.5 Miles	193,543 (59.4)	76,631 (61.1)	73,611 (59.5)
12.5–49.99 Miles	81,700 (25.1)	31,632 (25.2)	31,196 (25.2)
50–249.99 Miles	18,479 (5.7)	6,756 (5.4)	7,411 (6)
250 + Miles	2,333 (0.7)	1,002 (0.8)	953 (0.8)
**Medicaid Expansion State**			
Non-Expansion States	119,806 (36.7)	46,745 (37.2)	47,050 (38)
January 2014 Expansion States	96,782 (29.7)	37,610 (30)	35,443 (28.7)
Early Expansion States (2010–2013)	54,611 (16.8)	21,513 (17.1)	20,713 (16.7)
Late Expansion States (after Jan.2014)	43,085 (13.2)	15,792 (12.6)	15,912 (12.9)
**Charlson-Deyo Score**			
0	224,807 (69)	87,480 (69.7)	86,440 (69.9)
1	67,920 (20.8)	26,204 (20.9)	25,541 (20.7)
2	20,811 (6.4)	7,690 (6.1)	7,399 (6)
3 +	12,465 (3.8)	4,134 (3.3)	4,290 (3.5)
Facility Factors			
**Facility Type**			
Academic	83,649 (25.7)	33,280 (26.5)	31,503 (25.5)
Community Cancer Program	39,003 (12)	16,168 (12.9)	14,943 (12.1)
Comprehensive Community Cancer Program	144,768 (44.4)	56,190 (44.8)	55,049 (44.5)
Integrated Network Cancer Program	46,864 (14.4)	16,022 (12.8)	17,623 (14.3)
**Facility Location**			
South Atlantic	68,872 (21.1)	28,997 (23.1)	26,679 (21.6)
East North Central	56,544 (17.3)	22,098 (17.6)	21,065 (17)
Middle Atlantic	47,552 (14.6)	18,017 (14.4)	16,785 (13.6)
Pacific	35,902 (11)	12,879 (10.3)	13,966 (11.3)
West South Central	29,338 (9)	10,567 (8.4)	12,063 (9.8)
East South Central	23,714 (7.3)	9,132 (7.3)	9,334 (7.5)
West North Central	23,056 (7.1)	7,443 (5.9)	8,584 (6.9)
New England	16,671 (5.1)	7,347 (5.9)	5,613 (4.5)
Mountain	12,635 (3.9)	5,180 (4.1)	5,029 (4.1)
Treatment Details			
**Year of Diagnosis**			
2010–2012	119,032 (36.5)	55,187 (44)	45,863 (37.1)
2013–2015	122,914 (37.7)	47,932 (38.2)	46,654 (37.7)
2016–2017	84,057 (25.8)	22,389 (17.8)	31,153 (25.2)
**Operative Approach**			
Open	140,353 (43.1)	59,326 (47.3)	63,294 (51.2)
Laparoscopic	165,567 (50.8)	59,465 (47.4)	53,381 (43.2)
Robotic	20,083 (6.2)	6,717 (5.4)	6,995 (5.7)
**Tumor Location**			
Right/Transverse Colon	184,394 (56.6)	69,063 (55)	68,046 (55)
Left/Sigmoid Colon	132,868 (40.8)	52,857 (42.1)	51,831 (41.9)
Colon, not specified	8,741 (2.7)	3,588 (2.9)	3,793 (3.1)

^a^percentages may not add up to 100% due to missing data

### Domain 1: Clinical stage at presentation

Among 125,508 patients with known clinical and pathologic stage, 32% were diagnosed with clinical stage III or IV at presentation. Unadjusted comparisons of outcomes for all domains can be found in Additional file 1. After adjustment for other patient demographics, facility factors, year of diagnosis, and tumor location, Black (OR 1.09, *p* < 0.01), Hispanic/Spanish (OR 1.11 *p* < 0.01), and Southeast Asian (OR 1.39, *p* < 0.01) patients had significantly higher odds than White patients to present with clinical stage III/IV (Table 2).

**Table 2:** Differences in clinical stage at presentation and timing of surgery, by Race/Ethnicity

	**Domain 1:** **Clinical Stage at Presentation**		**Domain 2:** **Timing of Surgery**						
Race/Ethnicity	**aOR (Clinical Stage III/IV)**	***p*** **-value**	**aOR (> 42 day delay)**	***p*** **-value**	**aOR (Path Stage III/IV)**	***p*** **-value**		**aOR (Upstaged)**	***p*** **-value**
White	1.00	(ref)	1.00	(ref)	1.00	(ref)		1.00	(ref)
Black	1.09 (1.05–1.14)	< 0.01	1.33 (1.26–1.41)	< 0.01	1.05 (1.02–1.08)	< 0.01		0.97 (0.91, 1.02)	0.31
Hispanic/Spanish	1.11 (1.05–1.18)	< 0.01	1.32 (1.22–1.43)	< 0.01	1.05 (1.01–1.09)	0.01		1.03 (0.95, 1.13)	0.47
Southeast Asian	1.39 (1.20–1.60)	< 0.01	1.21 (0.99–1.47)	0.07	1.37 (1.26–1.49)	< 0.01		1.17 (0.94, 1.45)	0.16
East Asian	1.05 (0.93–1.19)	0.43	0.90 (0.76–1.08)	0.26	1.27 (1.19–1.36)	< 0.01		1.43 (1.22, 1.68)	< 0.01
South Asian	0.94 (0.76–1.18)	0.61	0.98 (0.72–1.34)	0.90	0.98 (0.86–1.11)	0.73		0.92 (0.67, 1.26)	0.61
AIAE	1.19 (0.95–1.48)	0.13	1.36 (1.00–1.87)	0.05	0.96 (0.83–1.10)	0.53		0.95 (0.67, 1.34)	0.76
NHOPI	1.22 (0.85–1.76)	0.28	1.04 (0.62–1.74)	0.89	1.32 (1.08–1.61)	0.01		1.34 (0.82, 2.21)	0.24

*AIAE*  American Indian, Aleutian, and Eskimo, *NHOPI*  Native Hawaiian and Other Pacific Islander

### Domain 2: Timing of surgery

Among all patients who received surgery, the average wait between diagnosis and first surgical procedure was 15 days (median of 4 days) and 10% of patients waited ≥ 42 days. After adjustment for other patient demographics, facility factors, year of diagnosis, tumor location, and clinical stage, Black (OR 1.33, *p* < 0.01), Hispanic/Spanish (OR 1.32, *p* < 0.01), and AIAE (OR 1.36, *p* = 0.05) patients had higher odds of a surgical delay of ≥ 42 days than White patients (Table 2).

A total of 39% of patients who received surgery as their primary intervention had pathologic stage III or IV. After adjustment for other patient demographics, facility factors, year of diagnosis, tumor location and clinical stage, Black (OR 1.05, *p* < 0.01), Hispanic/Spanish (OR 1.05, *p* = 0.02), Southeast Asian (OR 1.37, *p* < 0.01), East Asian (OR 1.27, *p* = 0.05), and NHOPI (OR 1.32, *p* < 0.01) patients had higher odds of pathologic stage III or IV than White patients. Only East Asian patients (OR 1.43, *p* < 0.01) had higher odds of being upstaged from a lower clinical stage at presentation to a higher pathologic stage than White patients (Table 2).

### Domain 3: Access to minimally invasive surgery

MIS techniques were used for 57% of surgical patients (51% laparoscopic, 6% robotic). After adjustment for other patient demographics, facility factors, year of diagnosis, and tumor location, Hispanic/Spanish (OR 1.08, *p* < 0.01), East Asian (OR 1.21, *p* < 0.01), and South Asian (OR 1.21, *p* = 0.01) patients were more likely to have surgery with an MIS technique than White patients. Black (OR 0.89, *p* < 0.01) and AIAE (OR 0.69, *p* = 0.04) patients were less likely to have robotic surgery than White patients (Table 3).

**Table 3:** Differences in access to minimally invasive surgery and post-surgical outcomes, by Race/Ethnicity

	**Domain 3:** **Access to Minimally Invasive Surgery**					**Domain 4:** **Post-operative Outcomes**						
Race/Ethnicity	**aOR (MIS)**	***p*** **-value**	**aOR (Robotic)**	***p*** **-value**	**aOR (Length of Stay > 7d)**	***p*** **-value**	**aOR (30-day readmission)**	***p*** **-value**	**aOR (30-day mortality)**	***p*** **-value**	**aOR (Composite)**	***p*** **-value**
White	1.00	(ref)	1.00	(ref)	1.00	(ref)	1.00	(ref)	1.00	(ref)	1.00	(ref)
Black	1.00 (0.97–1.03)	0.94	0.89 (0.85–0.95)	< 0.01	1.30 (1.26–1.34)	< 0.01	1.08 (1.02–1.14)	0.01	0.97 (0.90–1.05)	0.51	1.29 (1.25–1.32)	< 0.01
Hispanic/Spanish	1.08 (1.04–1.12)	< 0.01	1.04 (0.97–1.12)	0.25	0.95 (0.91–1.00)	0.05	0.97 (0.90–1.05)	0.48	0.76 (0.67–0.86)	< 0.01	0.94 (0.90–0.98)	< 0.01
Southeast Asian	1.00 (0.91–1.10)	0.98	1.13 (0.95–1.34)	0.16	0.88 (0.78–0.98)	0.02	1.03 (0.82–1.30)	0.78	0.61 (0.44–0.87)	0.01	0.89 (0.80–0.98)	0.03
East Asian	1.21 (1.12–1.31)	< 0.01	1.22 (1.06–1.39)	< 0.01	0.67 (0.61–0.74)	< 0.01	1.14 (0.96–1.35)	0.15	0.72 (0.56–0.92)	0.01	0.73 (0.67–0.80)	< 0.01
South Asian	1.21 (1.05–1.40)	0.01	1.16 (0.92–1.46)	0.22	0.89 (0.75–1.05)	0.20	1.01 (0.75–1.36)	0.94	0.83 (0.51–1.36)	0.46	0.94 (0.81–1.01)	0.46
AIAE	0.91 (0.78–1.06)	0.22	0.69 (0.48–0.98)	0.04	1.09 (0.92–1.30)	0.30	1.61 (1.24–2.09)	< 0.01	1.19 (0.80–1.76)	0.39	1.21 (1.03–1.41)	0.02
NHOPI	0.93 (0.75–1.16)	0.55	1.17 (0.81–1.69)	0.39	0.92 (0.71–1.19)	0.53	1.46 (0.93–2.31)	0.10	0.94 (0.46–1.92)	0.87	0.96 (0.75–1.23)	0.76

*AIAE* American Indian, Aleutian, and Eskimo, *NHOPI*  Native Hawaiian and Other Pacific Islander

### Domain 4: Post-operative outcomes

Among surgical patients, 22% had post-procedure hospital LOS of greater than 7 days, 5% were readmitted within 30 days of discharge, and 3% died within 30 days of discharge. A quarter of surgical patients had at least one of these poor outcomes. After adjustment for other patient demographics, facility factors, year of diagnosis, tumor location, pathologic stage, and operative approach, Hispanic/Spanish (OR 0.94, *p* < 0.01), Southeast Asian (OR 0.89, *p* = 0.03), and East Asian (OR 0.73, *p* < 0.01) patients were less likely to have at least one of these outcomes than White patients. Black (OR 1.29, *p* < 0.01) and AIAE (OR 1.21, *p* = 0.02) patients were more likely to have at least one of these outcomes than White patients (Table 3).

### Domain 5: Utilization of chemotherapy

A total of 79% of surgical patients with pathologic stage III or IV were recommended adjuvant chemotherapy. After adjustment for other patient demographics, facility factors, year of diagnosis, tumor location, operative approach, and post-operative complications, Southeast Asian (OR 1.45, *p* < 0.01), East Asian (OR 1.35, *p* < 0.01), and NHOPI (OR 1.73, *p* = 0.03) patients were more likely than White patients to be recommended chemotherapy (Table 4). Chemotherapy was administered to 87% of surgical patients for whom it was recommended. In our adjusted model, Black (OR 1.24, *p* < 0.01) and NHOPI (OR 2.18, *p* = 0.01) were more likely than White patients to have a delay in chemotherapy initiation greater than 90 days. These results remained largely unchanged when only surgical patients with pathologic stage III were examined (Additional file 2).

**Table 4:** Differences in access to chemotherapy for surgical patients with pathologic stage III and IV, by Race/Ethnicity

	**Domain 5:** **Utilization of Chemotherapy**									
Race/Ethnicity	**aOR** **(Recommended)**	***p*** **-value**	**aOR** **(Administered)**	***p*** **-value**	**aOR** **(Omitted)**	***p*** **-value**	**aOR** **(Too Sick/Died)**	***p*** **-value**	**aOR** **(Delay > 90 days)**	***p*** **-value**
White	1.00	(ref)	1.00	(ref)	1.00	(ref)	1.00	(ref)	1.00	(ref)
Black	1.00 (0.95–1.05)	0.98	0.92 (0.86–0.99)	0.03	1.12 (1.05–1.19)	< 0.01	1.12 (1.00–1.25)	0.05	1.24 (1.12–1.36)	< 0.01
Hispanic/Spanish	0.99 (0.92–1.06)	0.70	1.32 (1.18–1.47)	< 0.01	0.79 (0.72–0.87)	< 0.01	1.13 (0.94- 1.35)	0.19	1.14 (0.99–1.30)	0.06
Southeast Asian	1.45 (1.20–1.76)	< 0.01	1.12 (0.89–1.41)	0.32	0.84 (0.68–1.03)	0.09	0.70 (0.47–1.04)	0.08	1.17 (0.87–1.56)	0.30
East Asian	1.35 (1.16–1.58)	< 0.01	0.97 (0.81–1.17)	0.78	0.99 (0.83–1.16)	0.87	0.85 (0.63–1.16)	0.31	0.76 (0.56–1.03)	0.08
South Asian	1.00 (0.74–1.35)	0.98	0.91 (0.62–1.33)	0.62	1.10 (0.78–1.55)	0.59	0.90 (0.47–1.73)	0.76	0.85 (0.47–1.54)	0.60
AIAE	1.08 (0.81–1.45)	0.61	0.89 (0.62–1.29)	0.55	1.06 (0.76–1.49)	0.74	0.88 (0.47–1.63)	0.69	1.11 (0.65–1.91)	0.70
NHOPI	1.73 (1.04–2.76)	0.03	1.08 (0.63–1.86)	0.78	0.77 (0.46–1.29)	0.33	0.22 (0.06–0.80)	0.02	2.18 (1.26–3.75)	0.01

*AIAE*  American Indian, Aleutian, and Eskimo, *NHOPI*  Native Hawaiian and Other Pacific Islander

Furthermore, in our adjusted model, Black patients were more likely (OR 1.12, *p* < 0.01) and Hispanic/Spanish patients were less likely (OR 0.79, *p* < 0.01) than White patients to have chemotherapy omitted. Among patients for whom reasons for omission were available, Black patients were more likely (OR 1.12, *p* = 0.05) than White patients and NHOPI patients were less likely (OR 0.22, *p* = 0.02) than White patients to have chemotherapy omission due to being too sick or dying.

### Domain 6: Cumulative incidence of death

**Fig. 1:**
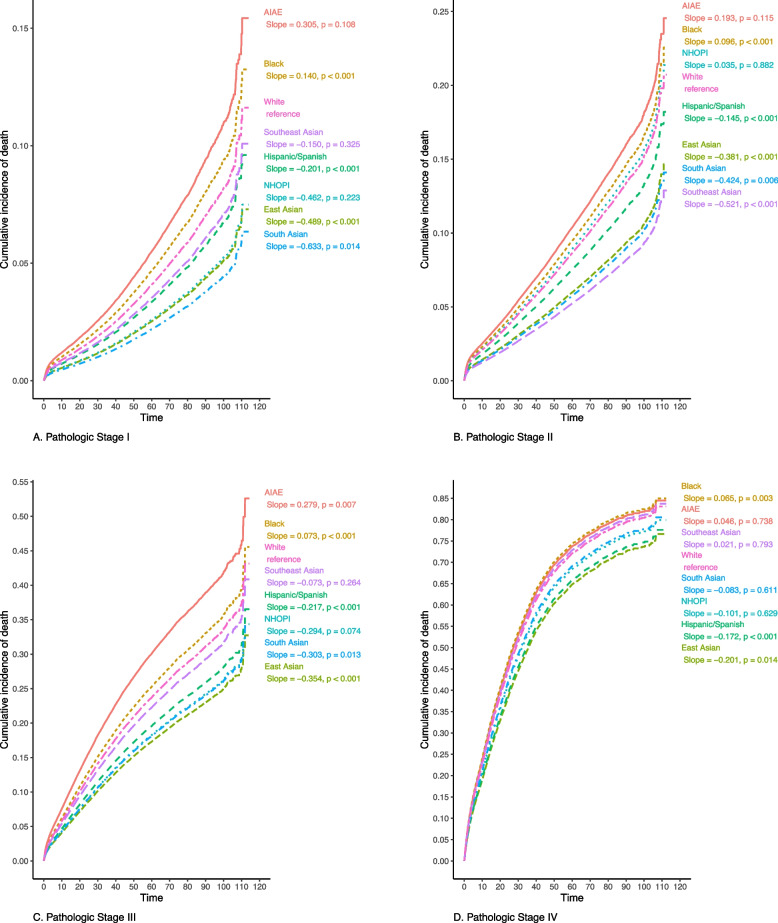
Cumulative incidence of death (adjusted for non-modifiable factors), by pathologic stage and Race/Ethnicity

After adjustment for non-modifiable factors (sex, age, geography, comorbidities, year of diagnosis, and tumor location), Black patients have higher cumulative incidence of death whereas East Asian and Hispanic/Spanish patients have lower incidence of death at every pathologic stage relative to White patients (*p* < 0.05, all; Fig. 1). Relative to White patients, South Asian patients have a lower incidence of death at pathologic stage I (*p* = 0.01), II (*p* = 0.01), and III (*p* = 0.01), with no significant difference for stage IV (*p* = 0.63). After further adjustment for modifiable factors (insurance, income quartile, education quartile, urban or rural county, facility type, distance traveled, and surgical approach), the difference in cumulative incidence of death for Black patients relative to White patients was no longer significant at any pathologic stage (Additional file 3).

Results were not materially changed when multiple imputation was applied to our analyses (data not shown).

## Discussion

In this large, national study, we examined differences in outcomes along the continuum of colon cancer management by race/ethnicity. In recognition of the complex, multifactorial nature of racial/ethnic attribution, we used the most detailed race/ethnicity categories available to us within the NCDB while accounting for sample size limitations. To the best of our knowledge, our study establishes the most granular and comprehensive picture of racial/ethnic differences across the colon cancer care continuum to-date. In so doing, our findings demonstrate differential outcomes of colon cancer presentation and management across the care continuum among different race/ethnicity groups, highlighting the need for deliberate and targeted interventions rather than a single overarching strategy for all groups.Among Asian patients, the most striking disparities were present in the early phase of the care continuum. Relative to White patients, Southeast Asian patients had higher odds of presenting with advanced clinical and pathologic stage, and East Asian patients had higher odds of advanced pathologic stage. This is concerning because presentation with more advanced disease negatively impacts prognosis [27–29]. Therefore, in these groups, focusing on timely diagnosis is critical. Once diagnosed, however, the odds of poor outcomes among Southeast and East Asian patients were not different from those of White patients. In fact, cumulative incidence of death for East Asian patients with pathologic stages I and II was the lowest of any racial/ethnic group examined, and for East Asian patients with pathologic stages III and IV was lower than most racial/ethnic groups examined. In contrast to Southeast Asian and East Asian groups, South Asian patients had no differences in outcomes relative to White patients across the care continuum except higher odds of MIS and lower cumulative incidence of death at pathologic stages I, II, and III, a finding consistent with other literature [2]. Existing literature on stage at presentation among Asian patients has been limited, inconclusive, and largely focused on “Asian” patients as a combined group [30–33]. Our study offers insights that may have been masked by the lack of granularity of prior work.

Importantly, we consider how our findings may reflect other work examining cultural attitudes toward cancer in Asian patients. Licquirish et al. noted a sense of embarrassment, fatalistic attitudes, religious and superstitious beliefs, association with cancer as deadly, and low health literacy about cancer among Southeast Asian and Chinese migrants [12]. Accordingly, Asian Americans have been found to have lower cancer screening rates than other groups [34–36] despite the documented association between screening and earlier-stage detection [37–39]. Foreign nativity, fewer years living in the U.S., and speaking a non-English language at home were all significantly associated with lower rates of colon cancer screening, independent of socioeconomic status and access to care among Southeast and East Asian Americans [14]. Language concordant, targeted educational interventions [40] and racial/ethnic-concordant educational instruction [41] have been found effective in some settings and should be broadly considered for at-risk groups to promote early presentation.

While cultural attitudes may play a role, they should be viewed in the context of systemic factors. Timely screening and early symptom identification rely on a comprehensive primary care model [42]. However, Asian Americans have the lowest odds of having a PCP of any racial/ethnic group [10]. Moreover, English-speaking Asian patients have reported lower rates of provider recommendations for screening than English-speaking White patients [43], a finding that may reflect systemic biases or differential access to high-quality providers and warrants future study.

This combination of factors may account, in part, for our finding that Southeast and East Asian patients had higher odds of advanced disease at presentation. However, we were limited in our study design in further classifying patients (i.e., based on nativity and primary language) and probing into cultural or systemic reasons for delayed presentation. Nonetheless, given our findings and the increasing rate of colorectal cancer incidence among some subgroups, addressing the gap in primary care may be considered as a strategy to bridge the disparity in colon cancer screening and presentation for Southeast Asian and East Asian patients [44, 45].

As with Asian patients, the most striking disparities among Hispanic/Spanish patients were in the early phase of the care continuum. Specifically, we found that Hispanic/Spanish patients presented with later-stage disease as reflected in higher odds of both advanced clinical and pathologic stage relative to White patients. This finding is consistent with other literature [33, 46], and may also be explained, in part, by the lower screening rates relative to White patients [46–48], particularly among patients who are Spanish-speaking [13], younger, and cancer free [17]. Hispanic/Spanish patients are also less likely than White patients to have an established PCP [10], which may similarly play a role in delayed presentation and thus more advanced stage. However, unlike other racial/ethnic minority groups, Hispanic/Spanish patients (both English and non-English speakers) did not report a lack of provider recommendation as a reason for not pursuing screening [43]. These differences between Hispanic/Spanish and Asian patients may, in part, explain why the odds of presenting with advanced clinical stage for Hispanic/Spanish patients relative to White patients (OR 1.11) were lower than that of Southeast Asian patients relative to White patients (OR 1.39) in our study*.* Nonetheless, our findings of higher odds of advanced-stage cancer in the setting of lower screening rates compared to White patients are concerning.

Our study also found that Hispanic/Spanish patients had more than 30% higher odds of surgical delay beyond 42 days than White patients. Our collective understanding of the implications of this finding is inconclusive. Multiple studies have found that colon cancer treatment delays (including surgery) were not associated with worse survival, a finding that has been attributed to improved quality and safety of resection and treatment due to sufficient time to complete proper preoperative evaluation and staging [49–51]. However, a study of patients with stage I and II colon cancer showed a 9% increase in 5-year mortality for every week delay beyond 42 days (with no difference in odds of death up to 41 days) [25]. This suggests there is a balance between sufficient time for a comprehensive workup (without impact on survival) and delay (which negatively impacts survival). It is reassuring that, despite increased odds of surgical delay beyond 42 days, we found a lower cumulative incidence of death for Hispanic/Spanish patients relative to White patients regardless of pathologic stage. Nonetheless, the independent difference in surgical delay between Hispanic/Spanish and White patients may reflect systemic bias [52] and should be addressed through strategies such as clear guidelines, blinded scheduling, and routine tracking of such differences. Similar to Southeast Asian and East Asian patients, beyond the early phase, Spanish/Hispanic patients had outcomes that were not different from those of White patients.

**Fig. 2:**
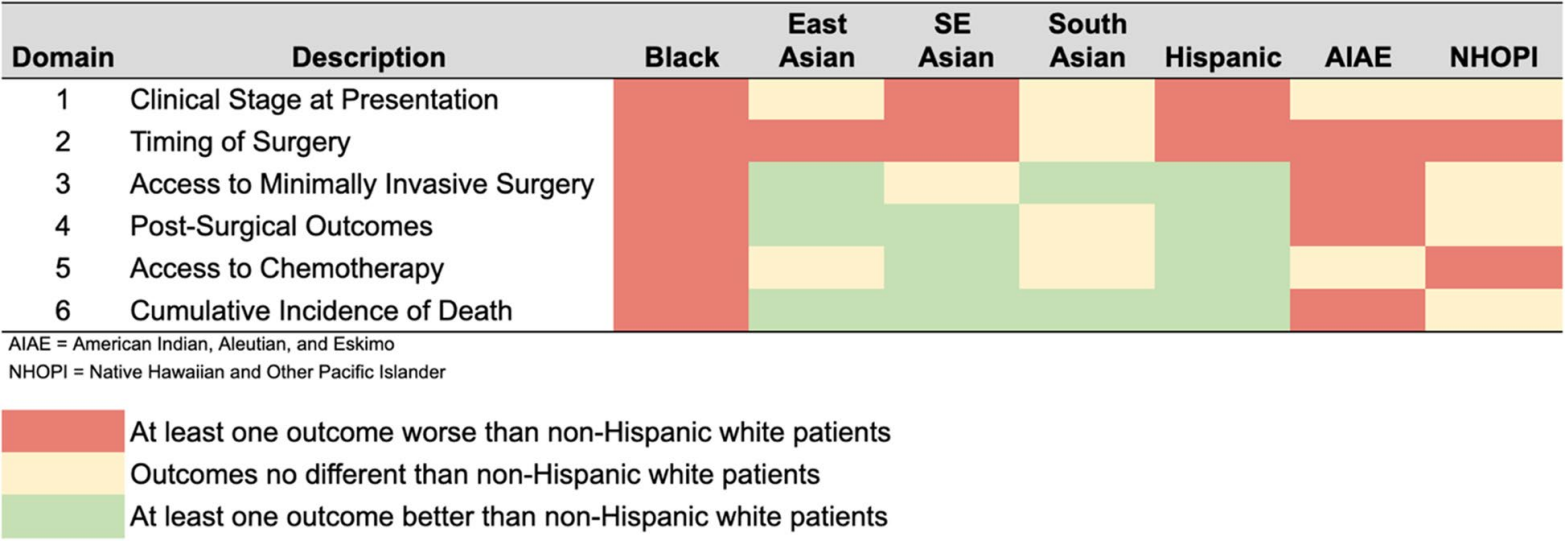
Summary of outcomes by Race/Ethnicity

Unlike findings for Asian and Hispanic/Spanish patients that demonstrated disparities in select domains of the care continuum, disparities affecting Black patients were seen across all six domains examined (Fig. 2). Relative to White patients, Black patients were found to have independently higher odds of presenting with advanced disease, surgical delays, non-robotic surgery, and post-operative complications; worse utilization of chemotherapy; and higher cumulative incidence of death. Although individual findings from our study are consistent with prior work [2, 8, 53–56], together, these findings are extremely concerning because they highlight the juxtaposition of colon cancer outcomes for Black patients relative to White patients vs. those of all other groups relative to White patients across all domains of the colon cancer care continuum.

The U.S. healthcare system carries a history of injustice toward Black patients. Experimentation on Black communities [57–59] and racial segregation of care delivery with inferior medical care [60] offer two distinct examples. Though such overt practices have been outlawed, disparities have persisted. Delays in care [61–64] and poorer outcomes [65–68] have been repeatedly shown for Black patients across healthcare settings. False provider beliefs about biological differences between Black and White patients, such as pain threshold, skin thickness, and brain size, have influenced how Black patients are treated within the healthcare system [69, 70].

It is in this broad context that we consider our findings related to colon cancer diagnosis and management. Black patients have lower odds of having a PCP [10] and a lower likelihood of receiving a provider recommendation for colon cancer screening [43], factors that may contribute to the lagging colorectal cancer screening rates among Black patients [39, 71] and increased odds of presenting with advanced disease, as seen in our study. In addition, persistent mistrust of the medical system [58, 72], understandably informed by the history of experimentation and perceived inequity of treatment, has been identified as a barrier to screening [11]. Moreover, despite recognition of concerning symptoms, Black patients have cited access to care (e.g., lack of a regular provider or delays in care) as a barrier to diagnosis [73]. Our study findings, which are based on a contemporary national dataset, reinforce the persistence of disparities in colon cancer outcomes (beginning at diagnosis) despite prior awareness raised and work done to date. Notably, while our study is consistent with others in that Black patients had worse access to robotic surgery over our study period [74, 75], the rapid adoption of robotic technology over the last decade [76] makes this finding difficult to interpret. However, as adoption of this technology persists, continued efforts should be made to understand potential disparities in access and ensure broad accessibility.

While targeted interventions may be appropriate for some groups, that our study identified disparities affecting Black patients across all six domains examined suggests that interventions across multiple medical specialties (e.g., primary care, medical oncology, colorectal surgery) and care settings (e.g., ancillary, inpatient, and outpatient services) are needed to achieve a major transformation of the current colon cancer screening and management paradigm for this group. Existing literature highlights the importance of establishing trust, increasing patient education, and enabling shared decision-making [11]—principles that can be employed in any clinical setting. While these are critical steps, evidence suggests that only a small portion of health disparities (e.g., differences in premature death) are attributed to the healthcare system [77]. Environmental factors are thought to account for the majority of colorectal cancer risk [78], rendering healthcare system-driven interventions necessary but insufficient. This is reinforced by our findings about cumulative incidence of death. Whereas significant differences between Black and White patients were seen when our analysis accounted for non-modifiable factors (e.g., age, sex), these differences were no longer significant when we also accounted for modifiable structural elements (e.g., insurance, income). Transformation not only within the healthcare system, but also at a societal level is needed.

Our findings should be viewed in the context of several limitations. First, our study population was limited to those patients who received surgery for their colon cancer. Assessment of disparities in access to surgery among colon cancer patients and reasons for situations when surgery was not performed is outside the scope of the present study and represents an opportunity for further exploration. Second, causes are impossible to ascertain from any retrospective study. However, our study presents as comprehensive a picture as possible about differences in colon cancer management by race/ethnicity and therefore offers critical insights as a starting point for further exploration. Third, our study considered and accounted for patient demographics, facility factors, year of diagnosis, stage, tumor location, operative approach, and post-operative complications, as applicable, in assessment of disparities within each domain. However, examining the relative effect of these factors on disparities within each domain is outside the scope of the present study and is reserved for future work. Fourth, though we tried to incorporate as much detail as possible, the race/ethnicity data from the NCDB is still limited. There is potential heterogeneity in how race and ethnicity are attributed by participating facilities and how often the best practice of self-identification takes place is unknown. Further, “Asian” (including East Asian, Southeast Asian, South Asian, and NHOPI) and “Hispanic/Spanish” patients represent diverse groups, each with origins from more than 30 countries. Each sub-group has a unique culture, which may differentially impact attitudes toward screening and presentation for symptoms. Similarly, we could not further classify Black patients into those with ancestors who were part of the colonial slave trade vs. those who either themselves or whose family members immigrated freely. This difference may affect both outcomes and potential root causes. Fifth, our results suggest that NHOPI patients had higher odds of having advanced pathologic stage, refusing chemotherapy, and experiencing chemotherapy delay relative to White patients, whereas AIAE patients had higher odds of surgical delays, non-robotic surgery, and post-surgical complications. The small sample size of these groups relative to other groups obviated further analysis, but should be the focus of future studies. Sixth, there are other factors, such as primary language, immigration status, and generational status, that may affect and explain the disparities seen. However, these factors are not captured in the NCDB, thus limiting our ability to account for them in the present study. Finally, our discussion focuses on potential elements explaining the observed disadvantages various patients groups face relative to non-Hispanic White patients, consistent with our aim to characterize racial/ethnic disparities in colon cancer care. However, our findings reveal several areas where various patient groups had better outcomes relative to non-Hispanic White patients. In-depth exploration of these areas is outside the scope of the present study but represents a future opportunity. Despite these limitations, our study offers important insights into big-picture differences and opportunities across the continuum of colon cancer care.

In conclusion, for colon cancer patients across the U.S., advanced stage at presentation is disproportionately experienced by non-White patients, and with varying influences among different racial/ethnic groups. While disparities for most racial/ethnic groups are limited to early phases of colon cancer management, disparities for Black patients are present across the entire care continuum. Identifying these differences is a key step to providing equitable care. Targeted interventions may be appropriate for some groups. However, major transformation – within and outside the healthcare system – is needed to address disparities experienced by Black patients.

## Supplementary Information

**Additional file 1.** Bivariate analysis results for Domains 1 & 2 Appendix 1a. Bivariate analysis results for Domains 1 & 2.

**Additional file 2.** Differences in access to chemotherapy for surgical patients with pathologic stage III, by Race/Ethnicity.

**Additional file 3.** Cumulative incidence of death (adjusted by non-modifiable and modifiable factors), by pathologic stage and race/ethnicity.

## Data Availability

The dataset supporting the conclusions of this article is available in the 2010–2017 National Cancer Database.

## Abbreviations

NCDB: National Cancer Database
AIAE: American Indian, Aleutian, and Eskimo
NHOPI: Native Hawaiian and other Pacific Islander
LOS: Length of stay
NCCN: National Comprehensive Cancer Network®
PCP: Primary care physician
