# Supplementary material for: Exploring the complexity and spectrum of racial/ethnic disparities in colon cancer management
Source: Int J Equity Health. 2023 Apr 14;22:68. doi: 10.1186/s12939-023-01883-w (PMC10105474; doi:10.1186/s12939-023-01883-w)
Supplement: Supplementary file 1 — Additional file 1. Bivariate analysis results for Domains 1 & 2 Appendix 1a. Bivariate analysis results for Domains 1 & 2. [file 12939_2023_1883_MOESM1_ESM.docx]

| **Appendix 1a. Bivariate analysis results for Domains 1 & 2** | | | | | | | | | | | | |
| --- | --- | --- | --- | --- | --- | --- | --- | --- | --- | --- | --- | --- |
|  |  | **Domain 1. Clinical Stage** |  |  |  | **Domain 2. Timing of Surgery** |  |  |  |  |  |  |
|  |  | **I/II** | **III/IV** | **p-value** |  | **Delay > 42 days** | | |  | **Pathologic Stage** | | |
| **Characteristic** |  |  |  |  |  | **No** | **Yes** | **p-value** |  | **I/II** | **III/IV** | **p-value** |
| Patient Demographics |  |  |  |  |  |  |  |  |  |  |  |  |
| **Age Category** |  |  |  | <0.001 |  |  |  | <0.001 |  |  |  | <0.001 |
| 18-49 |  | 10.50% | 15.90% |  |  | 11.90% | 7.40% |  |  | 9.90% | 13.20% |  |
| 50-59 |  | 21.50% | 22.60% |  |  | 20.50% | 19.40% |  |  | 20.00% | 20.90% |  |
| 60-69 |  | 25.70% | 25.60% |  |  | 25.20% | 27.10% |  |  | 25.60% | 25.20% |  |
| 70-79 |  | 23.40% | 19.60% |  |  | 23.20% | 26.40% |  |  | 24.60% | 22.00% |  |
| 80+ |  | 18.90% | 16.30% |  |  | 19.20% | 19.70% |  |  | 19.80% | 18.60% |  |
| **Sex** |  |  |  | <0.001 |  |  |  | <0.001 |  |  |  | 0.31 |
| Female |  | 50.00% | 51.30% |  |  | 49.30% | 51.60% |  |  | 49.50% | 49.70% |  |
| Male |  | 50.00% | 48.70% |  |  | 50.70% | 48.40% |  |  | 50.50% | 50.30% |  |
| **Race/Ethnicity** |  |  |  | <0.001 |  |  |  | <0.001 |  |  |  | <0.001 |
| White |  | 77.20% | 73.10% |  |  | 77.30% | 70.80% |  |  | 77.60% | 75.00% |  |
| American Indian, Aleutian, and Eskimo |  | 0.30% | 0.40% |  |  | 0.30% | 0.40% |  |  | 0.30% | 0.30% |  |
| East Asian |  | 1.20% | 1.20% |  |  | 1.20% | 1.40% |  |  | 1.20% | 1.40% |  |
| Native Hawaiian and Other Pacific Islander |  | 0.10% | 0.20% |  |  | 0.2 | 0.20% |  |  | 0.10% | 0.20% |  |
| Other Asian |  | 0.80% | 0.80% |  |  | 0.9 | 1.10% |  |  | 0.90% | 1.00% |  |
| South Asian |  | 0.40% | 0.40% |  |  | 0.4 | 0.40% |  |  | 0.40% | 0.40% |  |
| Southeast Asian |  | 0.80% | 0.90% |  |  | 0.8 | 1.20% |  |  | 0.80% | 1.00% |  |
| Black |  | 12.80% | 15.70% |  |  | 12.40% | 16.10% |  |  | 12.20% | 13.60% |  |
| Hispanic/Spanish |  | 5.80% | 6.70% |  |  | 5.90% | 7.80% |  |  | 5.90% | 6.50% |  |
| Other |  | 0.60% | 0.60% |  |  | 0.60% | 0.60% |  |  | 0.60% | 0.60% |  |
| **Payor** |  |  |  | <0.001 |  |  |  | <0.001 |  |  |  | <0.001 |
| Commercial |  | 38.20% | 38.50% |  |  | 37.90% | 32.50% |  |  | 36.80% | 37.90% |  |
| Medicaid |  | 5.80% | 9.20% |  |  | 5.80% | 7.20% |  |  | 5.40% | 6.80% |  |
| Medicare |  | 51.90% | 45.80% |  |  | 51.90% | 56.20% |  |  | 54.10% | 50.20% |  |
| Other Government |  | 1.00% | 1.10% |  |  | 0.90% | 1.10% |  |  | 0.90% | 1.00% |  |
| Uninsured |  | 3.00% | 5.50% |  |  | 3.40% | 3.00% |  |  | 2.80% | 4.10% |  |
| **Income Quartile** |  |  |  | <0.001 |  |  |  | <0.001 |  |  |  | <0.001 |
| Q1 |  | 17.90% | 19.90% |  |  | 18.00% | 19.60% |  |  | 17.40% | 19.00% |  |
| Q2 |  | 26.60% | 26.00% |  |  | 26.80% | 25.60% |  |  | 26.80% | 26.50% |  |
| Q3 |  | 23.40% | 23.60% |  |  | 23.30% | 23.70% |  |  | 23.10% | 23.50% |  |
| Q4 |  | 32.10% | 30.60% |  |  | 32.00% | 31.10% |  |  | 32.70% | 31.10% |  |
| **High School Degree Quartile** |  |  |  | <0.001 |  |  |  | <0.001 |  |  |  | <0.001 |
| Q1 |  | 22.60% | 21.40% |  |  | 23.20% | 20.00% |  |  | 23.30% | 22.20% |  |
| Q2 |  | 26.60% | 27.20% |  |  | 26.20% | 27.10% |  |  | 26.10% | 26.70% |  |
| Q3 |  | 32.60% | 31.40% |  |  | 32.40% | 31.80% |  |  | 32.70% | 31.70% |  |
| Q4 |  | 18.10% | 19.90% |  |  | 18.10% | 21.10% |  |  | 17.90% | 19.40% |  |
| **County** |  |  |  | <0.001 |  |  |  | <0.001 |  |  |  | <0.001 |
| Metro |  | 85.90% | 84.80% |  |  | 85.60% | 86.80% |  |  | 86.00% | 85.30% |  |
| Urban |  | 12.50% | 13.40% |  |  | 12.60% | 11.60% |  |  | 12.30% | 12.80% |  |
| Rural |  | 1.60% | 1.80% |  |  | 1.80% | 1.60% |  |  | 1.70% | 1.90% |  |
| **Distance Travelled** |  |  |  | <0.001 |  |  |  | <0.001 |  |  |  | <0.001 |
| <12.5 Miles |  | 65.80% | 61.70% |  |  | 65.80% | 61.30% |  |  | 65.50% | 65.00% |  |
| 12.5-49.99 Miles |  | 27.40% | 28.70% |  |  | 27.50% | 28.90% |  |  | 27.70% | 27.60% |  |
| 50-249.99 Miles |  | 5.90% | 8.10% |  |  | 6.00% | 8.80% |  |  | 6.00% | 6.50% |  |
| 250+ Miles |  | 0.80% | 1.40% |  |  | 0.80% | 1.00% |  |  | 0.70% | 0.80% |  |
| **Medicaid Expansion State** |  |  |  | <0.001 |  |  |  | <0.001 |  |  |  | <0.001 |
| Non-Expansion States |  | 37.20% | 38.40% |  |  | 38.90% | 33.80% |  |  | 37.40% | 39.50% |  |
| January 2014 Expansion States |  | 32.20% | 30.80% |  |  | 30.60% | 32.70% |  |  | 31.40% | 29.80% |  |
| Early Expansion States (2010-2013) |  | 17.40% | 17.30% |  |  | 16.70% | 19.90% |  |  | 17.40% | 17.40% |  |
| Late Expansion States (after Jan.2014) |  | 13.30% | 13.50% |  |  | 13.80% | 13.50% |  |  | 13.80% | 13.40% |  |
| **Charlson-Deyo Score** |  |  |  | <0.001 |  |  |  | <0.001 |  |  |  | <0.001 |
| 0 |  | 69.90% | 73.80% |  |  | 69.40% | 63.40% |  |  | 68.00% | 69.90% |  |
| 1 |  | 20.50% | 18.20% |  |  | 20.70% | 22.80% |  |  | 21.20% | 20.70% |  |
| 2 |  | 6.10% | 5.10% |  |  | 6.30% | 7.90% |  |  | 6.70% | 6.00% |  |
| 3+ |  | 3.50% | 3.00% |  |  | 3.60% | 5.90% |  |  | 4.10% | 3.50% |  |
| Facility Factors |  |  |  |  |  |  |  |  |  |  |  |  |
| **Facility Type** |  |  |  | <0.001 |  |  |  | <0.001 |  |  |  | 0.22 |
| Academic |  | 28.50% | 33.40% |  |  | 25.20% | 38.80% |  |  | 26.70% | 26.40% |  |
| Community Cancer Program |  | 13.40% | 12.10% |  |  | 12.80% | 8.80% |  |  | 12.30% | 12.50% |  |
| Comprehensive Community Cancer Program |  | 45.10% | 41.40% |  |  | 47.00% | 37.80% |  |  | 46.10% | 46.20% |  |
| Integrated Network Cancer Program |  | 13.00% | 13.00% |  |  | 15.00% | 14.60% |  |  | 14.90% | 14.80% |  |
| **Facility Location** |  |  |  | <0.001 |  |  |  | <0.001 |  |  |  | <0.001 |
| South Atlantic |  | 23.30% | 22.20% |  |  | 22.20% | 22.40% |  |  | 21.70% | 22.40% |  |
| East North Central |  | 18.40% | 17.50% |  |  | 18.30% | 17.60% |  |  | 18.20% | 17.70% |  |
| Middle Atlantic |  | 16.00% | 15.90% |  |  | 14.60% | 18.00% |  |  | 15.80% | 14.10% |  |
| Pacific |  | 10.40% | 10.90% |  |  | 10.70% | 13.90% |  |  | 11.20% | 11.70% |  |
| West South Central |  | 8.10% | 10.00% |  |  | 9.30% | 9.00% |  |  | 8.80% | 10.10% |  |
| East South Central |  | 7.00% | 7.20% |  |  | 7.90% | 4.90% |  |  | 7.30% | 7.80% |  |
| West North Central |  | 6.00% | 6.90% |  |  | 7.60% | 5.40% |  |  | 7.40% | 7.20% |  |
| New England |  | 6.80% | 5.30% |  |  | 5.30% | 5.50% |  |  | 5.70% | 4.70% |  |
| Mountain |  | 4.00% | 4.10% |  |  | 4.10% | 3.30% |  |  | 3.80% | 4.20% |  |
| Treatment Details |  |  |  |  |  |  |  |  |  |  |  |  |
| **Year of Diagnosis** |  |  |  | <0.001 |  |  |  | <0.001 |  |  |  | <0.001 |
| 2010-2012 |  | 43.50% | 37.50% |  |  | 36.80% | 31.50% |  |  | 36.10% | 37.10% |  |
| 2013-2015 |  | 38.10% | 38.80% |  |  | 37.80% | 37.60% |  |  | 38.10% | 37.70% |  |
| 2016-2017 |  | 18.50% | 23.70% |  |  | 25.40% | 31.00% |  |  | 25.80% | 25.20% |  |
| **Tumor Location** |  |  |  |  |  |  |  | <0.001 |  |  |  | <0.001 |
| Right/Transverse Colon |  | 51.20% | 46.30% |  |  | 57.00% | 53.90% |  |  | 57.70% | 55.00% |  |
| Left/Sigmoid Colon |  | 45.90% | 42.90% |  |  | 40.30% | 43.60% |  |  | 40.00% | 41.90% |  |
| Colon, not specified |  | 2.90% | 10.80% |  |  | 2.70% | 2.50% |  |  | 2.40% | 3.10% |  |

| **Appendix 1b. Bivariate analysis results for Domains 3 & 4** | | | | | | | | | | | | | | | | | | | | | | | | |  |
| --- | --- | --- | --- | --- | --- | --- | --- | --- | --- | --- | --- | --- | --- | --- | --- | --- | --- | --- | --- | --- | --- | --- | --- | --- | --- |
|  |  | **Domain 3. Access to Minimally Invasive Surgery** | | | | | | |  | **Domain 4. Postsurgical Outcomes** | | | | | |  | |  |  |  |  |  |  |  | |
|  |  | **Laparoscopic or Robotic Surgery** | | |  | **Robotic Surgery** | | |  | **Length of Stay>7d** | | |  | **30day readmission** | |  | | **30day mortality** | | |  | **Composite** | | | |
| **Characteristic** |  | **No** | **Yes** | **p-value** |  | **No** | **Yes** | **p-value** |  | **No** | **Yes** | **pvalue** |  | **No** | **Yes** | **pvalue** |  | **No** | **Yes** | **pvalue** |  | **No** | **Yes** | **pvalue** | |
| Patient Demographics |  |  |  |  |  |  |  |  |  |  |  |  |  |  |  |  |  |  |  |  |  |  |  |  | |
| **Age Category** |  |  |  | <0.001 |  |  |  | <0.001 |  |  |  | <0.001 |  |  |  | <0.001 |  |  |  | <0.001 |  |  |  | <0.001 | |
| 18-49 |  | 10.70% | 12.10% |  |  | 11.40% | 12.10% |  |  | 12.40% | 8.00% |  |  | 11.50% | 10.90% |  |  | 11.60% | 2.00% |  |  | 12.60% | 8.20% |  | |
| 50-59 |  | 18.50% | 21.90% |  |  | 20.20% | 23.50% |  |  | 22.00% | 14.70% |  |  | 20.60% | 18.20% |  |  | 20.90% | 6.70% |  |  | 22.30% | 15.00% |  | |
| 60-69 |  | 24.60% | 26.00% |  |  | 25.30% | 27.70% |  |  | 26.20% | 22.50% |  |  | 25.50% | 23.70% |  |  | 25.60% | 16.10% |  |  | 26.40% | 22.40% |  | |
| 70-79 |  | 23.80% | 23.30% |  |  | 23.50% | 23.10% |  |  | 22.80% | 25.90% |  |  | 23.40% | 24.40% |  |  | 23.40% | 26.60% |  |  | 22.80% | 25.60% |  | |
| 80+ |  | 22.40% | 16.70% |  |  | 19.60% | 13.60% |  |  | 16.50% | 28.90% |  |  | 19.00% | 22.80% |  |  | 18.60% | 48.50% |  |  | 15.90% | 28.70% |  | |
| **Sex** |  |  |  | <0.001 |  |  |  | <0.001 |  |  |  | <0.001 |  |  |  | 0.15 |  |  |  | <0.001 |  |  |  | <0.001 | |
| Female |  | 48.60% | 50.40% |  |  | 49.40% | 52.00% |  |  | 48.80% | 52.30% |  |  | 49.50% | 50.10% |  |  | 49.30% | 52.30% |  |  | 48.80% | 51.90% |  | |
| Male |  | 51.40% | 49.60% |  |  | 50.60% | 48.00% |  |  | 51.20% | 47.70% |  |  | 50.50% | 49.90% |  |  | 50.70% | 47.70% |  |  | 51.20% | 48.10% |  | |
| **Race/Ethnicity** |  |  |  | <0.001 |  |  |  | <0.001 |  |  |  | <0.001 |  |  |  | <0.001 |  |  |  | <0.001 |  |  |  | <0.001 | |
| White |  | 76.20% | 76.70% |  |  | 76.50% | 76.20% |  |  | 76.80% | 75.60% |  |  | 76.70% | 75.60% |  |  | 76.60% | 82.40% |  |  | 76.70% | 75.90% |  | |
| American Indian, Aleutian, and Eskimo |  | 0.30% | 0.30% |  |  | 0.30% | 0.20% |  |  | 0.30% | 0.40% |  |  | 0.30% | 0.50% |  |  | 0.30% | 0.30% |  |  | 0.30% | 0.40% |  | |
| East Asian |  | 1.10% | 1.50% |  |  | 1.30% | 1.70% |  |  | 1.40% | 1.00% |  |  | 1.30% | 1.10% |  |  | 1.30% | 0.80% |  |  | 1.40% | 1.00% |  | |
| Native Hawaiian and Other Pacific Islander |  | 0.20% | 0.20% |  |  | 0.20% | 0.20% |  |  | 0.20% | 0.20% |  |  | 0.20% | 0.20% |  |  | 0.20% | 0.10% |  |  | 0.20% | 0.10% |  | |
| Other Asian |  | 0.80% | 1.10% |  |  | 0.90% | 1.20% |  |  | 1.00% | 0.60% |  |  | 0.90% | 0.70% |  |  | 0.90% | 0.40% |  |  | 1.00% | 0.70% |  | |
| South Asian |  | 0.30% | 0.50% |  |  | 0.40% | 0.60% |  |  | 0.40% | 0.30% |  |  | 0.40% | 0.40% |  |  | 0.40% | 0.20% |  |  | 0.40% | 0.30% |  | |
| Southeast Asian |  | 0.80% | 0.90% |  |  | 0.90% | 1.00% |  |  | 0.90% | 0.80% |  |  | 0.90% | 0.60% |  |  | 0.90% | 0.40% |  |  | 0.90% | 0.70% |  | |
| Black |  | 13.80% | 12.00% |  |  | 12.80% | 11.50% |  |  | 12.10% | 15.10% |  |  | 12.60% | 14.60% |  |  | 12.80% | 11.10% |  |  | 12.00% | 14.80% |  | |
| Hispanic/Spanish |  | 6.00% | 6.30% |  |  | 6.10% | 6.70% |  |  | 6.30% | 5.60% |  |  | 6.20% | 6.00% |  |  | 6.00% | 3.90% |  |  | 6.30% | 5.60% |  | |
| Other |  | 0.50% | 0.60% |  |  | 0.60% | 0.70% |  |  | 0.60% | 0.50% |  |  | 0.60% | 0.40% |  |  | 0.60% | 0.30% |  |  | 0.60% | 0.50% |  | |
| **Payor** |  |  |  | <0.001 |  |  |  | <0.001 |  |  |  | <0.001 |  |  |  | <0.001 |  |  |  | <0.001 |  |  |  | <0.001 | |
| Commercial |  | 32.80% | 40.90% |  |  | 37.00% | 44.10% |  |  | 40.90% | 25.10% |  |  | 37.90% | 29.90% |  |  | 38.10% | 14.70% |  |  | 41.50% | 25.70% |  | |
| Medicaid |  | 6.50% | 5.50% |  |  | 6.00% | 5.20% |  |  | 5.70% | 7.00% |  |  | 5.90% | 7.00% |  |  | 5.80% | 3.90% |  |  | 5.70% | 6.80% |  | |
| Medicare |  | 55.30% | 50.00% |  |  | 52.50% | 48.30% |  |  | 49.20% | 63.30% |  |  | 52.00% | 57.90% |  |  | 51.70% | 78.00% |  |  | 48.70% | 62.80% |  | |
| Other Government |  | 0.90% | 1.00% |  |  | 1.00% | 0.80% |  |  | 0.90% | 0.90% |  |  | 1.00% | 1.00% |  |  | 0.90% | 0.70% |  |  | 1.00% | 0.90% |  | |
| Uninsured |  | 4.50% | 2.50% |  |  | 3.50% | 1.70% |  |  | 3.20% | 3.80% |  |  | 3.30% | 4.20% |  |  | 3.40% | 2.60% |  |  | 3.20% | 3.80% |  | |
| **Income Quartile** |  |  |  | <0.001 |  |  |  | <0.001 |  |  |  | <0.001 |  |  |  | <0.001 |  |  |  | <0.001 |  |  |  | <0.001 | |
| Q1 |  | 20.80% | 15.90% |  |  | 18.20% | 15.50% |  |  | 17.10% | 20.80% |  |  | 17.90% | 20.80% |  |  | 18.00% | 20.60% |  |  | 17.10% | 20.80% |  | |
| Q2 |  | 26.50% | 26.70% |  |  | 26.60% | 26.70% |  |  | 26.60% | 26.40% |  |  | 26.60% | 26.60% |  |  | 26.70% | 26.70% |  |  | 26.70% | 26.50% |  | |
| Q3 |  | 25.10% | 21.70% |  |  | 23.40% | 20.80% |  |  | 22.60% | 24.20% |  |  | 23.20% | 23.50% |  |  | 23.10% | 26.00% |  |  | 22.90% | 24.10% |  | |
| Q4 |  | 27.60% | 35.60% |  |  | 31.80% | 36.90% |  |  | 33.60% | 28.60% |  |  | 32.30% | 29.10% |  |  | 32.20% | 26.70% |  |  | 33.30% | 28.60% |  | |
| **High School Degree Quartile** |  |  |  | <0.001 |  |  |  | <0.001 |  |  |  | <0.001 |  |  |  | <0.001 |  |  |  | <0.001 |  |  |  | <0.001 | |
| Q1 |  | 19.70% | 25.30% |  |  | 22.70% | 26.20% |  |  | 23.80% | 20.50% |  |  | 23.00% | 20.70% |  |  | 23.00% | 19.40% |  |  | 23.70% | 20.50% |  | |
| Q2 |  | 28.10% | 24.90% |  |  | 26.40% | 24.20% |  |  | 25.60% | 27.90% |  |  | 26.20% | 27.40% |  |  | 26.30% | 28.00% |  |  | 25.80% | 27.70% |  | |
| Q3 |  | 31.90% | 32.60% |  |  | 32.30% | 32.80% |  |  | 32.60% | 31.60% |  |  | 32.30% | 32.30% |  |  | 32.30% | 33.80% |  |  | 32.40% | 31.80% |  | |
| Q4 |  | 20.30% | 17.10% |  |  | 18.60% | 16.70% |  |  | 18.00% | 20.00% |  |  | 18.40% | 19.60% |  |  | 18.50% | 18.80% |  |  | 18.00% | 19.90% |  | |
| **County** |  |  |  | <0.001 |  |  |  | <0.001 |  |  |  | <0.001 |  |  |  | 0.003 |  |  |  | <0.001 |  |  |  | <0.001 | |
| Metro |  | 83.60% | 87.50% |  |  | 85.60% | 88.70% |  |  | 86.00% | 84.70% |  |  | 85.80% | 85.10% |  |  | 85.90% | 83.40% |  |  | 86.20% | 84.70% |  | |
| Urban |  | 14.40% | 11.00% |  |  | 12.60% | 10.00% |  |  | 12.20% | 13.30% |  |  | 12.50% | 12.90% |  |  | 12.40% | 14.20% |  |  | 12.20% | 13.30% |  | |
| Rural |  | 2.00% | 1.60% |  |  | 1.80% | 1.30% |  |  | 1.70% | 2.00% |  |  | 1.70% | 2.10% |  |  | 1.70% | 2.40% |  |  | 1.70% | 2.00% |  | |
| **Distance Travelled** |  |  |  | <0.001 |  |  |  | <0.001 |  |  |  | <0.001 |  |  |  | <0.001 |  |  |  | <0.001 |  |  |  | <0.001 | |
| <12.5 Miles |  | 65.70% | 65.10% |  |  | 65.60% | 62.50% |  |  | 65.00% | 66.80% |  |  | 65.20% | 67.70% |  |  | 65.70% | 69.00% |  |  | 64.80% | 66.90% |  | |
| 12.5-49.99 Miles |  | 27.20% | 27.90% |  |  | 27.40% | 30.00% |  |  | 28.10% | 25.70% |  |  | 27.70% | 26.10% |  |  | 27.40% | 24.00% |  |  | 28.20% | 25.80% |  | |
| 50-249.99 Miles |  | 6.30% | 6.20% |  |  | 6.20% | 6.70% |  |  | 6.20% | 6.70% |  |  | 6.30% | 5.80% |  |  | 6.10% | 6.10% |  |  | 6.10% | 6.50% |  | |
| 250+ Miles |  | 0.80% | 0.80% |  |  | 0.80% | 0.80% |  |  | 0.70% | 0.80% |  |  | 0.80% | 0.40% |  |  | 0.70% | 0.80% |  |  | 0.80% | 0.80% |  | |
| **Medicaid Expansion State** |  |  |  | <0.001 |  |  |  | <0.001 |  |  |  | <0.001 |  |  |  | <0.001 |  |  |  | 0.008 |  |  |  | <0.001 | |
| Non-Expansion States |  | 38.90% | 37.50% |  |  | 38.10% | 38.70% |  |  | 37.10% | 37.70% |  |  | 38.10% | 39.40% |  |  | 38.00% | 39.20% |  |  | 38.10% | 38.10% |  | |
| January 2014 Expansion States |  | 30.90% | 30.70% |  |  | 30.90% | 29.30% |  |  | 30.80% | 31.50% |  |  | 30.50% | 33.90% |  |  | 30.90% | 30.40% |  |  | 30.50% | 31.70% |  | |
| Early Expansion States (2010-2013) |  | 16.50% | 18.10% |  |  | 17.30% | 18.40% |  |  | 18.20% | 16.60% |  |  | 17.60% | 12.70% |  |  | 17.40% | 16.20% |  |  | 17.80% | 16.10% |  | |
| Late Expansion States (after Jan.2014) |  | 13.70% | 13.70% |  |  | 13.70% | 13.60% |  |  | 13.80% | 14.30% |  |  | 13.70% | 14.00% |  |  | 13.80% | 14.10% |  |  | 13.60% | 14.20% |  | |
| **Charlson-Deyo Score** |  |  |  | <0.001 |  |  |  | <0.001 |  |  |  | <0.001 |  |  |  | <0.001 |  |  |  | <0.001 |  |  |  | <0.001 | |
| 0 |  | 68.00% | 69.70% |  |  | 68.80% | 71.30% |  |  | 70.60% | 61.60% |  |  | 69.30% | 61.90% |  |  | 69.10% | 56.00% |  |  | 71.40% | 61.80% |  | |
| 1 |  | 21.40% | 20.40% |  |  | 20.90% | 19.50% |  |  | 20.30% | 23.50% |  |  | 20.70% | 23.00% |  |  | 21.30% | 25.20% |  |  | 20.00% | 23.30% |  | |
| 2 |  | 6.80% | 6.10% |  |  | 6.40% | 5.40% |  |  | 5.70% | 9.00% |  |  | 6.20% | 9.10% |  |  | 6.30% | 10.80% |  |  | 5.50% | 9.00% |  | |
| 3+ |  | 3.90% | 3.80% |  |  | 3.80% | 3.70% |  |  | 3.30% | 5.90% |  |  | 3.70% | 6.10% |  |  | 3.30% | 8.00% |  |  | 3.10% | 5.90% |  | |
| Facility Factors |  |  |  |  |  |  |  |  |  |  |  |  |  |  |  |  |  |  |  |  |  |  |  |  | |
| **Facility Type** |  |  |  | <0.001 |  |  |  | <0.001 |  |  |  | <0.001 |  |  |  | 0.48 |  |  |  | <0.001 |  |  |  | <0.001 | |
| Academic |  | 24.90% | 27.90% |  |  | 26.40% | 30.10% |  |  | 26.70% | 26.00% |  |  | 26.40% | 26.90% |  |  | 26.60% | 20.00% |  |  | 26.90% | 25.70% |  | |
| Community Cancer Program |  | 15.70% | 9.90% |  |  | 12.70% | 7.90% |  |  | 11.70% | 12.90% |  |  | 12.40% | 12.20% |  |  | 12.50% | 15.20% |  |  | 12.20% | 12.90% |  | |
| Comprehensive Community Cancer Program |  | 45.80% | 46.30% |  |  | 46.20% | 44.20% |  |  | 46.50% | 46.20% |  |  | 46.20% | 45.80% |  |  | 46.00% | 49.90% |  |  | 45.90% | 46.50% |  | |
| Integrated Network Cancer Program |  | 13.60% | 15.90% |  |  | 14.70% | 17.70% |  |  | 15.20% | 14.90% |  |  | 14.90% | 15.10% |  |  | 14.90% | 14.90% |  |  | 14.90% | 14.90% |  | |
| **Facility Location** |  |  |  | <0.001 |  |  |  | <0.001 |  |  |  | <0.001 |  |  |  | <0.001 |  |  |  | <0.001 |  |  |  | <0.001 | |
| South Atlantic |  | 22.60% | 21.40% |  |  | 21.70% | 25.00% |  |  | 21.00% | 21.70% |  |  | 22.00% | 19.90% |  |  | 21.90% | 23.10% |  |  | 22.00% | 21.60% |  | |
| East North Central |  | 19.50% | 16.80% |  |  | 18.00% | 18.00% |  |  | 17.80% | 18.50% |  |  | 18.00% | 19.10% |  |  | 18.00% | 18.70% |  |  | 17.80% | 18.50% |  | |
| Middle Atlantic |  | 13.40% | 16.40% |  |  | 15.00% | 17.30% |  |  | 15.40% | 15.40% |  |  | 14.80% | 17.60% |  |  | 15.20% | 14.00% |  |  | 15.00% | 15.60% |  | |
| Pacific |  | 10.90% | 11.80% |  |  | 11.40% | 12.10% |  |  | 12.10% | 11.00% |  |  | 11.80% | 5.60% |  |  | 11.40% | 10.20% |  |  | 11.80% | 10.20% |  | |
| West South Central |  | 10.50% | 8.40% |  |  | 9.40% | 8.90% |  |  | 9.10% | 9.30% |  |  | 9.20% | 12.10% |  |  | 9.30% | 8.40% |  |  | 9.20% | 9.80% |  | |
| East South Central |  | 8.70% | 6.60% |  |  | 7.60% | 6.10% |  |  | 7.30% | 8.40% |  |  | 7.60% | 7.80% |  |  | 7.50% | 8.80% |  |  | 7.30% | 8.30% |  | |
| West North Central |  | 6.60% | 7.90% |  |  | 7.50% | 5.60% |  |  | 7.50% | 7.30% |  |  | 7.40% | 7.40% |  |  | 7.30% | 7.40% |  |  | 7.30% | 7.40% |  | |
| New England |  | 4.20% | 6.20% |  |  | 5.50% | 3.00% |  |  | 5.70% | 4.70% |  |  | 5.30% | 6.50% |  |  | 5.40% | 4.70% |  |  | 5.40% | 4.90% |  | |
| Mountain |  | 3.50% | 4.40% |  |  | 4.00% | 4.10% |  |  | 4.10% | 3.60% |  |  | 4.00% | 3.90% |  |  | 4.00% | 4.70% |  |  | 4.10% | 3.80% |  | |
| Treatment Details |  |  |  |  |  |  |  |  |  |  |  |  |  |  |  |  |  |  |  |  |  |  |  |  | |
| **Year of Diagnosis** |  |  |  | <0.001 |  |  |  | <0.001 |  |  |  | <0.001 |  |  |  | <0.001 |  |  |  | <0.001 |  |  |  | <0.001 | |
| 2010-2012 |  | 45.80% | 29.50% |  |  | 38.00% | 13.30% |  |  | 34.70% | 40.80% |  |  | 36.40% | 38.90% |  |  | 41.90% | 46.10% |  |  | 35.10% | 40.70% |  | |
| 2013-2015 |  | 35.40% | 39.50% |  |  | 37.70% | 37.00% |  |  | 37.90% | 36.90% |  |  | 37.80% | 36.90% |  |  | 43.20% | 41.30% |  |  | 37.90% | 37.10% |  | |
| 2016-2017 |  | 18.80% | 31.00% |  |  | 24.20% | 49.70% |  |  | 27.40% | 22.20% |  |  | 25.90% | 24.20% |  |  | 14.80% | 12.60% |  |  | 27.00% | 22.20% |  | |
| **Tumor Location** |  |  |  | <0.001 |  |  |  | <0.001 |  |  |  | <0.001 |  |  |  | <0.001 |  |  |  | <0.001 |  |  |  | <0.001 | |
| Right/Transverse Colon |  | 56.90% | 56.30% |  |  | 57.00% | 49.40% |  |  | 56.50% | 57.50% |  |  | 56.50% | 58.80% |  |  | 56.20% | 61.30% |  |  | 56.10% | 57.90% |  | |
| Left/Sigmoid Colon |  | 39.90% | 41.40% |  |  | 40.20% | 48.70% |  |  | 41.10% | 39.20% |  |  | 40.90% | 38.20% |  |  | 41.10% | 34.00% |  |  | 41.40% | 38.80% |  | |
| Colon, not specified |  | 3.20% | 2.30% |  |  | 2.70% | 1.90% |  |  | 2.40% | 3.30% |  |  | 2.70% | 3.00% |  |  | 2.60% | 4.70% |  |  | 2.50% | 3.30% |  | |

| **Appendix 1c. Bivariate analysis results for Domain 5** | | | | | | | | | | | | | | | | | |
| --- | --- | --- | --- | --- | --- | --- | --- | --- | --- | --- | --- | --- | --- | --- | --- | --- | --- |
|  |  | **Domain 5. Access to Chemotherapy** |  |  |  |  |  |  |  |  |  |  |  |  |  |  |  |
|  |  | **Chemo Recommended** | | |  | **Chemo Administered** | | |  | **Chemo Omitted** | | |  | **Chemo Delay>90days** | | |  |
| **Characteristic** |  | **No** | **Yes** | **pvalue** |  | **No** | **Yes** | **pvalue** |  | **No** | **Yes** | **pvalue** |  | **No** | **Yes** | **pvalue** |  |
| Patient Demographics |  |  |  |  |  |  |  |  |  |  |  |  |  |  |  |  |  |
| **Age Category** |  |  |  | <0.001 |  |  |  | <0.001 |  |  |  | <0.001 |  |  |  | <0.001 |  |
| 18-49 |  | 6.20% | 15.10% |  |  | 4.70% | 16.80% |  |  | 16.80% | 4.00% |  |  | 17.10% | 11.80% |  |  |
| 50-59 |  | 10.60% | 23.60% |  |  | 10.90% | 25.70% |  |  | 25.70% | 9.40% |  |  | 25.90% | 23.20% |  |  |
| 60-69 |  | 16.40% | 27.60% |  |  | 19.80% | 28.80% |  |  | 28.80% | 18.20% |  |  | 28.70% | 31.40% |  |  |
| 70-79 |  | 21.00% | 22.30% |  |  | 29.50% | 21.30% |  |  | 21.30% | 28.70% |  |  | 21.20% | 23.40% |  |  |
| 80+ |  | 45.70% | 11.30% |  |  | 35.10% | 7.30% |  |  | 7.30% | 39.80% |  |  | 7.10% | 10.20% |  |  |
| **Sex** |  |  |  | <0.001 |  |  |  | <0.001 |  |  |  | <0.001 |  |  |  | 0.39 |  |
| Female |  | 45.70% | 50.80% |  |  | 46.20% | 51.40% |  |  | 51.40% | 45.50% |  |  | 51.30% | 52.00% |  |  |
| Male |  | 54.30% | 49.20% |  |  | 53.80% | 48.60% |  |  | 48.60% | 54.50% |  |  | 48.70% | 48.00% |  |  |
| **Race/Ethnicity** |  |  |  | <0.001 |  |  |  | <0.001 |  |  |  | <0.001 |  |  |  | <0.001 |  |
| White |  | 77.50% | 74.40% |  |  | 77.50% | 74.30% |  |  | 74.30% | 77.90% |  |  | 75.00% | 67.40% |  |  |
| American Indian, Aleutian, and Eskimo |  | 0.30% | 0.30% |  |  | 0.40% | 0.30% |  |  | 0.30% | 0.30% |  |  | 0.30% | 0.40% |  |  |
| East Asian |  | 1.10% | 1.50% |  |  | 1.70% | 1.50% |  |  | 1.50% | 1.50% |  |  | 1.40% | 1.30% |  |  |
| Native Hawaiian and Other Pacific Islander |  | 0.10% | 0.20% |  |  | 0.20% | 0.20% |  |  | 0.20% | 0.20% |  |  | 0.20% | 0.40% |  |  |
| Other Asian |  | 0.80% | 1.00% |  |  | 0.90% | 1.00% |  |  | 1.00% | 0.80% |  |  | 1.00% | 1.00% |  |  |
| South Asian |  | 0.30% | 0.40% |  |  | 0.30% | 0.40% |  |  | 0.40% | 0.30% |  |  | 0.40% | 0.30% |  |  |
| Southeast Asian |  | 0.70% | 1.10% |  |  | 0.90% | 1.20% |  |  | 1.20% | 0.80% |  |  | 1.00% | 1.50% |  |  |
| Black |  | 12.40% | 13.90% |  |  | 13.10% | 13.90% |  |  | 13.90% | 13.20% |  |  | 13.70% | 18.80% |  |  |
| Hispanic/Spanish |  | 6.10% | 6.60% |  |  | 4.70% | 6.70% |  |  | 6.70% | 4.70% |  |  | 6.40% | 8.40% |  |  |
| Other |  | 0.60% | 0.50% |  |  | 0.50% | 0.50% |  |  | 0.50% | 0.40% |  |  | 0.50% | 0.60% |  |  |
| **Payor** |  |  |  | <0.001 |  |  |  | <0.001 |  |  |  | <0.001 |  |  |  | <0.001 |  |
| Commercial |  | 21.30% | 42.40% |  |  | 19.60% | 46.20% |  |  | 46.20% | 17.00% |  |  | 47.00% | 32.40% |  |  |
| Medicaid |  | 5.20% | 7.20% |  |  | 5.90% | 7.40% |  |  | 7.40% | 5.60% |  |  | 7.10% | 11.80% |  |  |
| Medicare |  | 69.80% | 44.90% |  |  | 69.80% | 41.00% |  |  | 41.00% | 73.30% |  |  | 40.50% | 48.00% |  |  |
| Other Government |  | 0.90% | 1.00% |  |  | 0.80% | 1.00% |  |  | 1.00% | 0.70% |  |  | 1.00% | 0.90% |  |  |
| Uninsured |  | 2.80% | 4.50% |  |  | 4.00% | 4.50% |  |  | 4.50% | 3.30% |  |  | 4.40% | 6.80% |  |  |
| **Income Quartile** |  |  |  | <0.001 |  |  |  | <0.001 |  |  |  | <0.001 |  |  |  | <0.001 |  |
| Q1 |  | 20.10% | 18.70% |  |  | 19.70% | 18.50% |  |  | 18.50% | 19.80% |  |  | 18.40% | 23.00% |  |  |
| Q2 |  | 26.50% | 26.50% |  |  | 26.40% | 26.60% |  |  | 26.60% | 26.80% |  |  | 26.70% | 26.00% |  |  |
| Q3 |  | 24.30% | 23.20% |  |  | 24.60% | 23.00% |  |  | 23.00% | 24.40% |  |  | 23.30% | 23.40% |  |  |
| Q4 |  | 29.10% | 31.60% |  |  | 29.20% | 31.90% |  |  | 31.90% | 29.00% |  |  | 31.60% | 27.70% |  |  |
| **High School Degree Quartile** |  |  |  | <0.001 |  |  |  | <0.001 |  |  |  | <0.001 |  |  |  | <0.001 |  |
| Q1 |  | 21.40% | 22.40% |  |  | 21.20% | 22.60% |  |  | 22.60% | 21.20% |  |  | 22.90% | 18.50% |  |  |
| Q2 |  | 27.40% | 26.50% |  |  | 26.50% | 26.40% |  |  | 26.40% | 26.60% |  |  | 26.40% | 27.90% |  |  |
| Q3 |  | 31.40% | 31.90% |  |  | 33.40% | 31.80% |  |  | 31.80% | 33.50% |  |  | 32.10% | 29.70% |  |  |
| Q4 |  | 19.80% | 19.30% |  |  | 18.90% | 19.10% |  |  | 19.10% | 18.70% |  |  | 18.60% | 23.90% |  |  |
| **County** |  |  |  | 0.92 |  |  |  | <0.001 |  |  |  | <0.001 |  |  |  | 0.003 |  |
| Metro |  | 85.40% | 85.30% |  |  | 83.90% | 85.40% |  |  | 85.40% | 84.30% |  |  | 85.20% | 86.10% |  |  |
| Urban |  | 12.80% | 12.90% |  |  | 14.10% | 12.70% |  |  | 12.70% | 13.70% |  |  | 12.90% | 12.30% |  |  |
| Rural |  | 1.90% | 1.80% |  |  | 2.00% | 1.80% |  |  | 1.80% | 2.00% |  |  | 1.90% | 1.60% |  |  |
| **Distance Travelled** |  |  |  | <0.001 |  |  |  | <0.001 |  |  |  | <0.001 |  |  |  | <0.001 |  |
| <12.5 Miles |  | 66.60% | 64.60% |  |  | 67.20% | 64.30% |  |  | 64.30% | 67.40% |  |  | 63.90% | 68.20% |  |  |
| 12.5-49.99 Miles |  | 25.20% | 28.20% |  |  | 25.70% | 28.80% |  |  | 28.80% | 25.40% |  |  | 29.20% | 25.60% |  |  |
| 50-249.99 Miles |  | 7.20% | 6.40% |  |  | 6.20% | 6.20% |  |  | 6.20% | 6.50% |  |  | 6.20% | 5.40% |  |  |
| 250+ Miles |  | 1.10% | 0.80% |  |  | 0.80% | 0.60% |  |  | 0.60% | 0.70% |  |  | 0.60% | 0.80% |  |  |
| **Medicaid Expansion State** |  |  |  | <0.001 |  |  |  | <0.001 |  |  |  | <0.001 |  |  |  | <0.001 |  |
| Non-Expansion States |  | 41.10% | 39.00% |  |  | 35.50% | 39.30% |  |  | 39.30% | 36.10% |  |  | 40.20% | 38.60% |  |  |
| January 2014 Expansion States |  | 28.30% | 30.10% |  |  | 31.10% | 30.10% |  |  | 30.10% | 30.80% |  |  | 30.20% | 28.50% |  |  |
| Early Expansion States (2010-2013) |  | 18.10% | 17.20% |  |  | 18.20% | 17.10% |  |  | 17.10% | 18.00% |  |  | 16.10% | 19.60% |  |  |
| Late Expansion States (after Jan.2014) |  | 12.50% | 13.60% |  |  | 15.20% | 13.50% |  |  | 13.50% | 15.10% |  |  | 13.60% | 13.20% |  |  |
| **Charlson-Deyo Score** |  |  |  | <0.001 |  |  |  | <0.001 |  |  |  | <0.001 |  |  |  | <0.001 |  |
| 0 |  | 63.20% | 71.60% |  |  | 63.40% | 72.90% |  |  | 72.90% | 59.80% |  |  | 73.10% | 67.80% |  |  |
| 1 |  | 22.60% | 20.10% |  |  | 22.70% | 19.70% |  |  | 19.70% | 23.70% |  |  | 19.70% | 21.70% |  |  |
| 2 |  | 8.50% | 5.30% |  |  | 8.30% | 4.90% |  |  | 4.90% | 9.70% |  |  | 4.80% | 6.50% |  |  |
| 3+ |  | 5.70% | 2.90% |  |  | 5.60% | 2.50% |  |  | 2.50% | 6.80% |  |  | 2.40% | 4.00% |  |  |
| Facility Factors |  |  |  |  |  |  |  |  |  |  |  |  |  |  |  |  |  |
| **Facility Type** |  |  |  | <0.001 |  |  |  | <0.001 |  |  |  | <0.001 |  |  |  | <0.001 |  |
| Academic |  | 24.60% | 26.90% |  |  | 23.10% | 27.40% |  |  | 27.40% | 23.40% |  |  | 27.00% | 29.70% |  |  |
| Community Cancer Program |  | 13.70% | 12.20% |  |  | 13.10% | 12.10% |  |  | 12.10% | 12.60% |  |  | 11.90% | 12.60% |  |  |
| Comprehansive Community Cancer Program |  | 47.10% | 45.90% |  |  | 48.60% | 45.70% |  |  | 45.70% | 48.40% |  |  | 46.00% | 42.10% |  |  |
| Integrated Network Cancer Program |  | 14.60% | 14.90% |  |  | 15.20% | 14.90% |  |  | 14.90% | 15.60% |  |  | 15.10% | 15.60% |  |  |
| **Facility Location** |  |  |  | <0.001 |  |  |  | <0.001 |  |  |  | <0.001 |  |  |  | <0.001 |  |
| South Atlantic |  | 22.70% | 22.30% |  |  | 19.10% | 22.70% |  |  | 22.70% | 19.60% |  |  | 23.30% | 24.00% |  |  |
| East North Central |  | 16.10% | 18.20% |  |  | 20.00% | 18.20% |  |  | 18.20% | 20.20% |  |  | 18.80% | 17.20% |  |  |
| Middle Atlantic |  | 13.90% | 14.00% |  |  | 14.40% | 13.70% |  |  | 13.70% | 14.10% |  |  | 13.20% | 13.60% |  |  |
| Pacific |  | 12.10% | 11.70% |  |  | 12.80% | 11.60% |  |  | 11.60% | 12.00% |  |  | 10.50% | 14.40% |  |  |
| West South Central |  | 11.40% | 9.70% |  |  | 8.20% | 9.50% |  |  | 9.50% | 8.30% |  |  | 9.20% | 10.40% |  |  |
| East South Central |  | 8.00% | 7.80% |  |  | 6.60% | 8.20% |  |  | 8.20% | 6.90% |  |  | 8.30% | 7.70% |  |  |
| West North Central |  | 6.90% | 7.30% |  |  | 8.50% | 7.30% |  |  | 7.30% | 8.80% |  |  | 7.60% | 4.70% |  |  |
| New England |  | 4.50% | 4.80% |  |  | 5.50% | 4.70% |  |  | 4.70% | 5.80% |  |  | 4.80% | 4.40% |  |  |
| Mountain |  | 4.30% | 4.20% |  |  | 4.90% | 4.10% |  |  | 4.10% | 4.20% |  |  | 4.20% | 3.50% |  |  |
| Treatment Details |  |  |  |  |  |  |  |  |  |  |  |  |  |  |  |  |  |
| **Year of Diagnosis** |  |  |  | <0.001 |  |  |  | 0.43 |  |  |  | 0.094 |  |  |  | <0.001 |  |
| 2010-2012 |  | 39.00% | 36.60% |  |  | 36.40% | 36.70% |  |  | 36.70% | 36.70% |  |  | 35.90% | 40.00% |  |  |
| 2013-2015 |  | 36.60% | 38.10% |  |  | 38.60% | 38.00% |  |  | 38.00% | 38.60% |  |  | 38.20% | 37.60% |  |  |
| 2016-2017 |  | 24.40% | 25.40% |  |  | 25.00% | 25.40% |  |  | 25.40% | 24.70% |  |  | 25.90% | 22.40% |  |  |
| **Tumor Location** |  |  |  | <0.001 |  |  |  | <0.001 |  |  |  | <0.001 |  |  |  | 0.003 |  |
| Right/Transverse Colon |  | 62.40% | 53.10% |  |  | 60.50% | 52.00% |  |  | 52.00% | 61.90% |  |  | 52.20% | 50.90% |  |  |
| Left/Sigmoid Colon |  | 33.70% | 44.10% |  |  | 36.10% | 45.20% |  |  | 45.20% | 34.70% |  |  | 45.00% | 45.60% |  |  |
| Colon, not specified |  | 3.90% | 2.90% |  |  | 3.40% | 2.80% |  |  | 2.80% | 3.40% |  |  | 2.70% | 3.50% |  |  |
