# Supplementary material for: Exploring the complexity and spectrum of racial/ethnic disparities in colon cancer management
Source: Int J Equity Health. 2023 Apr 14;22:68. doi: 10.1186/s12939-023-01883-w (PMC10105474; doi:10.1186/s12939-023-01883-w)
Supplement: Supplementary file 2 — Additional file 2. Differences in access to chemotherapy for surgical patients with pathologic stage III, by Race/Ethnicity. [file 12939_2023_1883_MOESM2_ESM.docx]

| **Appendix 2.** Differences in access to chemotherapy for surgical patients with pathologic stage III, by Race/Ethnicity. | | | | | | | | | | | | | | | | |  |  |
| --- | --- | --- | --- | --- | --- | --- | --- | --- | --- | --- | --- | --- | --- | --- | --- | --- | --- | --- |
|  | |  |  | |  |  |  |  |  |  |  |  |  |  |  |  | |  |
|  | |  | **Domain 5: Access to Chemotherapy** | | | | | | | | | | | | | |  |  |
|  | |  |  | |  |  |  |  |  |  |  |  |  |  |  |  | |  |
| **Race/Ethnicity** | |  | **aOR  (Recommended)** | | **p-value** |  | **aOR  (Administered)** | **p-value** |  | **aOR  (Omitted)** | **p-value** |  | **aOR  (Too Sick/Died)** | **p-value** |  | **aOR  (Delay >90 days)** | | **p-value** |
| **White** | |  | 1.00 | | (ref) |  | 1.00 | (ref) |  | 1.00 | (ref) |  | 1.00 | (ref) |  | 1.00 | | (ref) |
| **Black** |  | | | 0.98 (0.92-1.04) | 0.44 |  | 0.88 (0.81-0.95) | <0.01 |  | 1.17 (1.09-1.26) | <0.01 |  | 1.12 (0.99-1.27) | 0.08 |  | 1.22 (1.09-1.37) | | <0.01 |
| **Hispanic/Spanish** | |  | 0.97 (0.89-1.06) | | 0.49 |  | 1.40 (1.24-1.59) | <0.01 |  | 0.75 (0.68-0.84) | <0.01 |  | 1.16 (0.95-1.42) | 0.14 |  | 1.16 (1.00-1.35) | | 0.05 |
| **Southeast Asian** | |  | 1.30 (1.04-1.63) | | 0.02 |  | 1.21 (0.93-1.58) | 0.15 |  | 0.78 (0.61-1.00) | 0.05 |  | 0.84 (0.52-1.34) | 0.46 |  | 1.27 (0.92-1.76) | | 0.14 |
| **East Asian** | |  | 1.30 (1.10-1.55) | | <0.01 |  | 0.94 (0.77-1.15) | 0.57 |  | 1.00 (0.83-1.20) | 0.99 |  | 0.81 (0.58-1.13) | 0.22 |  | 0.76 (0.54-1.07) | | 0.12 |
| **South Asian** | |  | 1.23 (0.86-1.77) | | 0.27 |  | 0.99 (0.64-1.52) | 0.96 |  | 1.00 (0.68-1.47) | 0.99 |  | 0.95 (0.45-2.01) | 0.89 |  | 0.78 (0.40-1.54) | | 0.48 |
| **AIAE** | |  | 1.08 (0.76-1.53) | | 0.68 |  | 0.78 (0.52-1.18) | 0.24 |  | 1.16 (0.80-1.70) | 0.44 |  | 0.95 (0.48-1.86) | 0.87 |  | 0.93 (0.47-1.86) | | 0.84 |
| **NAOPI** | |  | 2.04 (1.09-3.83) | | 0.03 |  | 1.11 (0.61-2.02) | 0.74 |  | 0.76 (0.43-1.35) | 0.35 |  | 0.31 (0.08-1.17) | 0.08 |  | 2.18 (1.19-4.00) | | 0.01 |
| AIAE = American Indian, Aleutian, and Eskimo | | | | | | | | | | | | | | | | |  |  |
| NHOPI + Native Hawaiian and Other Pacific Islander | | | | | | | | | | | | | | | | |  |  |
